# Analysis of clinical feature differences and influencing factors between children with peptic ulcer and healthy children

**DOI:** 10.3389/fped.2026.1849123

**Published:** 2026-10-14

**Authors:** Caiyan Chen, Huiying Qiu

**Affiliations:** Department of Paediatrics, Zhongshan Hospital of Xiamen University, Xiamen, China

**Keywords:** children, clinical features, influencing factors, peptic ulcer, retrospective study

## Abstract

This study conducted a retrospective analysis of clinical data from 92 children with peptic ulcer admitted to a tertiary hospital in the past 5 years and 92 healthy children in the same period, systematically exploring differences in clinical manifestations, laboratory indicators, lifestyle habits, and psychological status between the two groups. Multivariate logistic regression models were employed to identify independent risk factors for pediatric peptic ulcer. Results showed that the incidence of abdominal pain (82.6% vs. 15.2%), vomiting (63.0% vs. 8.7%), and melena (19.6% vs. 0%) was significantly higher in the patient group than in the healthy group (*P* < 0.05). Helicobacter pylori (Hp) infection rate (63.0% vs. 8.7%) and anemia incidence (45.7% vs. 12.0%) were also markedly elevated. Gastroscopy revealed that ulcers predominantly occurred in the duodenal bulb (68.5%) and gastric antrum (23.9%). Regarding lifestyle habits, the patient group exhibited significantly higher proportions of boarding school attendance (77.2% vs. 28.3%), high-sugar diet (56.5% vs. 28.3%), family history of digestive system diseases (30.4% vs. 8.7%), and psychological disorder prevalence (63.0% vs. 9.8%) compared to the control group (*P* < 0.01). Multivariate regression analysis confirmed that Hp infection (OR = 4.23), high-sugar diet (OR = 2.89), family history (OR = 3. 17), boarding school attendance (OR = 2.41), and psychological disorders (OR = 6. 11) were independent risk factors for pediatric peptic ulcer. This study provides evidence-based support for early identification and intervention of pediatric peptic ulcer, suggesting that clinical practice should strengthen screening for high-risk children and implement comprehensive interventions to improve prognosis.

## 1 Introduction

Pediatric peptic ulcer remains an important organic gastrointestinal disease in children. Recent studies and reviews have shown that peptic ulcer disease is closely associated with Helicobacter pylori infection, acid-related mucosal injury, and non-steroidal anti-inflammatory drug exposure, while endoscopy remains an important method for definitive diagnosis (1–3). In children, the clinical manifestations are often nonspecific, including recurrent abdominal pain, vomiting, hematemesis, and melena, which may overlap with functional gastrointestinal disorders, gastritis, or intestinal infection and may therefore lead to delayed diagnosis (3, 4). Although severe complications are relatively uncommon in children, gastrointestinal bleeding, perforation, and pyloric obstruction may occur in some cases, particularly when diagnosis or treatment is delayed, and these complications can adversely affect children's growth, development, and quality of life (2, 5).

Existing studies indicate that the pathogenesis of pediatric peptic ulcer is complex, involving multidimensional interactions including genetic susceptibility, environmental factors, lifestyle habits, and psychosocial factors. Helicobacter pylori (Hp) infection, a major etiological factor for peptic ulcer in adults, has been extensively validated in its pathogenic role in children. However, the natural history of Hp infection in children, eradication strategies, and associations with ulcer recurrence require further investigation (6, 7). Additionally, lifestyle factors such as high-sugar diet, boarding school life, and family history of digestive system diseases may influence ulcer development by affecting gastric acid secretion, mucosal barrier function, and intestinal flora composition (8–11). Although previous studies have investigated the clinical characteristics and risk factors of pediatric peptic ulcer, many of them were limited by small sample sizes, single-center data, or insufficient adjustment for multiple potential influencing factors. Therefore, further studies are needed to comprehensively evaluate clinical manifestations, laboratory indicators, lifestyle-related factors, family history, and psychological status in children with peptic ulcer.

The aim of this study was to compare clinical characteristics, laboratory findings, lifestyle-related factors, family history, and psychological status between children with peptic ulcer and healthy controls, and to identify factors independently associated with pediatric peptic ulcer using multivariate logistic regression analysis.

## 2 Objects and methods

### 2.1 Study subjects

In this study, 92 children diagnosed with peptic ulcer in the pediatric department of a tertiary hospital from January 2021 to January 2026 were selected as the case group. As this was a retrospective case-control study, the sample size was determined by the number of eligible children with complete clinical data during the predefined study period rather than by an *a priori* sample size calculation. To address the adequacy of the sample size, we performed a *post hoc* sample size assessment based on the differences in key variables between the two groups. Using a two-sided *α* of 0.05, a power of 80%, and a 1:1 case-control ratio, the minimum required sample size was lower than the actual sample size of 92 participants in each group. Therefore, the sample size of the present study was considered acceptable for the main between-group comparisons and multivariate logistic regression analysis. The inclusion criteria were as follows: (1) Diagnosed with gastric or duodenal ulcers via gastroscopy and meeting the “Gastroscopic Diagnostic Criteria for Chronic Gastritis and Peptic Ulcer in Children”; (2) No previous history of peptic ulcer; (3) No use of glucocorticoids, mucosal protectants, acid suppressants, or non-steroidal anti-inflammatory drugs within one month prior to the trial; (4) Aged between 8 and 14 years; (5) Complete clinical data without any missing information. During the same period, 92 healthy children who underwent routine physical examination in the same hospital were selected as the control group. The control children were recruited from the pediatric outpatients of the same hospital and were required to have no history of peptic ulcer, chronic gastrointestinal disease, infectious disease, severe chronic disease, or recent use of glucocorticoids, acid suppressants, mucosal protectants, or non-steroidal anti-inflammatory drugs. Age and sex distributions were compared between the two groups to evaluate baseline comparability.

### 2.2 Data collection

This study comprehensively collected and extracted multi-dimensional clinical information of the study subjects through a standardized electronic medical record system. Specifically, the demographic characteristics dimension included basic demographic variables such as age, gender, and boarding school status; the clinical manifestations dimension detailed the frequency and characteristics of symptoms related to peptic ulcer, such as abdominal pain, vomiting, and melena; the laboratory indicators dimension systematically collected objective laboratory data, including Helicobacter pylori status determined by the 13C-urea breath test, blood routine parameters, and fecal occult blood tests, to support diagnosis and assessment; the lifestyle habits dimension focused on dietary structure characteristics and family history of digestive system diseases, which are lifestyle-related factors; the psychological assessment dimension used the internationally recognized Screen for Child Anxiety Related Emotional Disorders (SCARED) to conduct quantitative scoring, with a score of ≥25 as the critical threshold for determining the presence of psychological disorders. All data extraction processes adhered to strict quality control procedures to ensure that the information was complete, accurate, and in line with the data collection norms for retrospective studies, providing a reliable data foundation for subsequent statistical analysis and identification of risk factors.

### 2.3 Study design

This study followed a rigorous retrospective case-control study process. First, children were screened through the hospital's electronic medical record system, and simultaneously, 92 healthy children undergoing physical examinations during the same period were included as the control group. Then, a standardized data extraction template was used to systematically collect relevant dimensional information from the electronic medical records. Finally, statistical analysis was conducted, and independent risk factors were screened using a multivariate logistic regression model

to ensure that the study process was scientific, standardized, and met the rigorous requirements for methodological description in SCI medical journals. See Figure 1.

**Figure 1:**
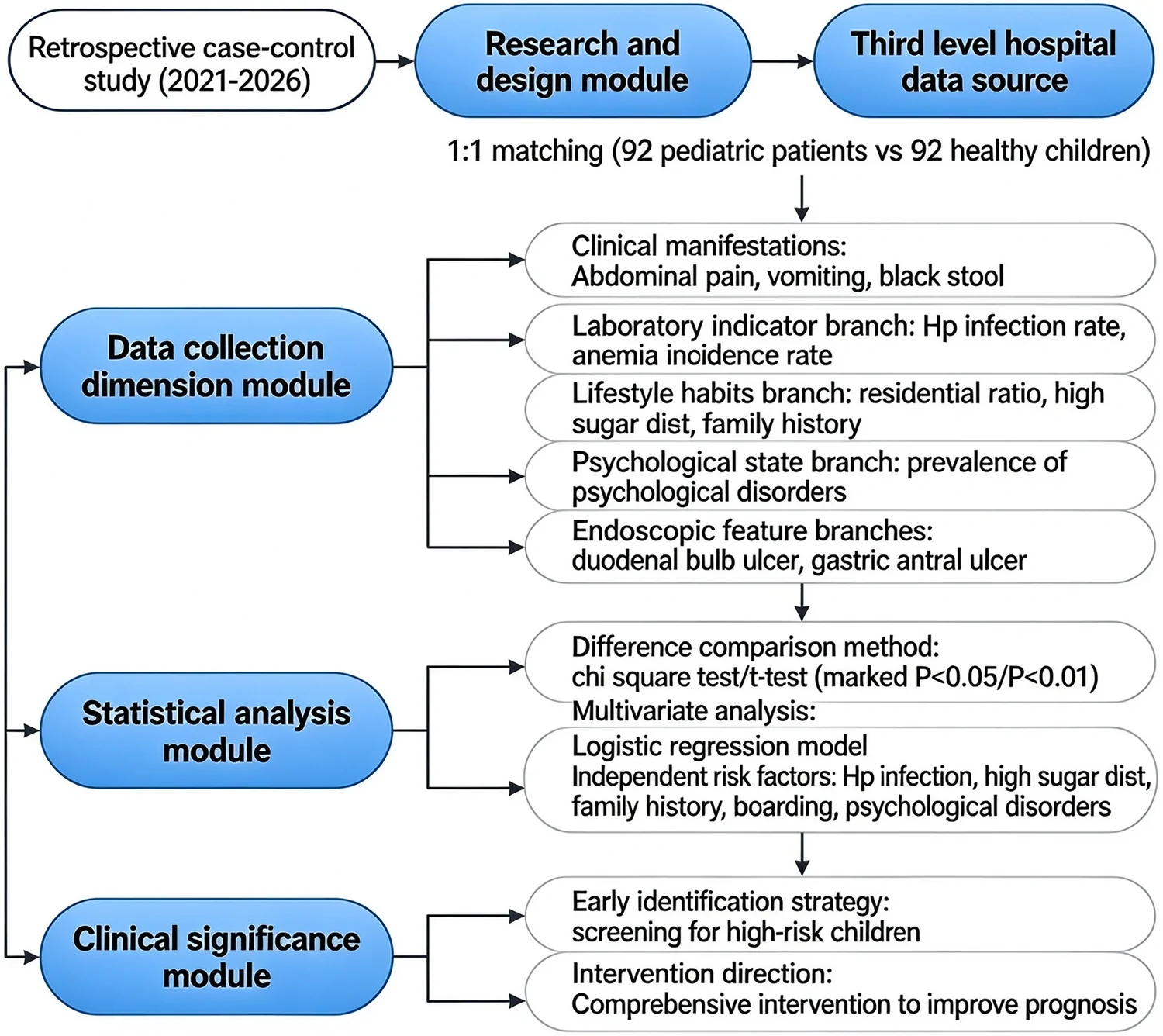
Flowchart of the study.

### 2.4 Statistical analysis

Data analysis was performed using SPSS 26.0 software, and image processing was carried out using GraphPad Prism 8 software. Continuous variables were expressed as mean ± standard deviation, and comparisons between groups were performed using the independent-samples t-test. Categorical variables were expressed as *n* (%), and comparisons between groups were performed using the chi-square test. When the expected frequency was less than 5, Fisher's exact test was applied. Considering the presence of multiple univariate comparisons, the Benjamini-Hochberg false discovery rate (FDR) method was used to adjust *P* values in the univariate analysis, in order to reduce the risk of false-positive findings. Variables with statistical significance after FDR adjustment, as well as variables considered clinically relevant, were considered as candidate variables for multivariate logistic regression analysis. Clinical manifestations such as abdominal pain, vomiting, and melena, as well as complication-related indicators such as anemia and positive fecal occult blood test, were analyzed in the univariate comparison but were not included as exposure variables in the risk-factor regression model because they may be consequences or diagnostic features of peptic ulcer rather than pre-exposure risk factors. A multivariate logistic regression model was used to identify independent factors associated with pediatric peptic ulcer. The enter method was used for model construction after considering statistical significance, clinical relevance, and collinearity among candidate variables. Model goodness of fit was assessed using the Hosmer-Lemeshow test. Multicollinearity was evaluated using tolerance and variance inflation factor (VIF), with VIF < 5 indicating no significant multicollinearity. Odds ratios (ORs) and 95% confidence intervals (CIs) were calculated. A two-sided *P* value < 0.05 was considered statistically significant.

### 2.5 Ethical statement

This study was reviewed and approved by the Ethics Committee of Zhongshan Hospital, Xiamen University (approval NO.: XMZSYYKY2026200). As this was a retrospective case-control study based on existing electronic medical records, and all data were anonymized before analysis, the requirement for written informed consent from the children or their legal guardians was waived by the Ethics Committee. The study was conducted in accordance with the Declaration of Helsinki and relevant institutional requirements. No potentially identifiable personal information was used or disclosed in this study.

## 3 Results

### 3.1 Demographic and baseline characteristics

The demographic characteristics, clinical manifestations, laboratory findings, lifestyle-related factors, family history, and psychological status of the two groups are summarized in Table 1. There were no significant differences in age or sex distribution between the peptic ulcer group and the healthy control group (*P* > 0.05), indicating that the two groups were generally comparable at baseline. Compared with the healthy control group, the peptic ulcer group had significantly higher proportions of abdominal pain, vomiting, melena, Helicobacter pylori infection, anemia, positive fecal occult blood test, boarding school attendance, high-sugar diet, family history of digestive system diseases, and psychological disorders (*P* < 0.05). In addition, the mean SCARED score was significantly higher in the peptic ulcer group than in the healthy control group (28.6 ± 8.4 vs. 14.2 ± 6.1, *P* < 0.001), suggesting that children with peptic ulcer had a higher level of anxiety-related emotional symptoms.

**Table 1:** Demographic and baseline characteristics and comparison of clinical manifestations, laboratory findings, lifestyle-related factors, family history, and psychological status between the two groups.

Clinical characteristic	Peptic ulcer group (*n* = 92)	Healthy control group (*n* = 92)	Test statistic (t/x^2^)	*P* value
Age (years)	11.6 ± 2.4	11.4 ± 2.6	0.493	0.622
Male	50 (54.3)	48 (52.2)	0.087	0.768
Abdominal pain	76 (82.6)	14 (15.2)	83.605	<0.001
Vomiting	58 (63.0)	8 (8.7)	59.065	<0.001
Melena	18 (19.6)	0 (0.0)	—	<0.001
Helicobacter pylori infection	58 (63.0)	8 (8.7)	59.065	<0.001
Anemia	42 (45.7)	11 (12.0)	25.468	<0.001
Positive fecal occult blood test	35 (38.0)	3 (3.3)	33.961	<0.001
Boarding school attendance	71 (77.2)	26 (28.3)	44.152	<0.001
High-sugar diet	52 (56.5)	26 (28.3)	15.044	<0.001
Family history of digestive system diseases	28 (30.4)	8 (8.7)	13.814	<0.001
SCARED score	28.6 ± 8.4	14.2 ± 6.1	13.309	<0.001
Psychological disorders	58 (63.0)	9 (9.8)	56.357	<0.001

Data are presented as mean ± standard deviation or *n* (%), as appropriate. SCARED, Screen for Child Anxiety Related Emotional Disorders. *P* values for the univariate comparisons were adjusted using the Benjamini–Hochberg false discovery rate method. Fisher's exact test was used when the expected cell frequency was less than 5.

### 3.2 Differences in clinical manifestations

The incidence rates of abdominal pain (82.6% vs. 15.2%), vomiting (63.0% vs. 8.7%), and melena (19.6% vs. 0%) in the child patient group were significantly higher than those in the healthy group (*P* < 0.05); see Figure 2.

**Figure 2:**
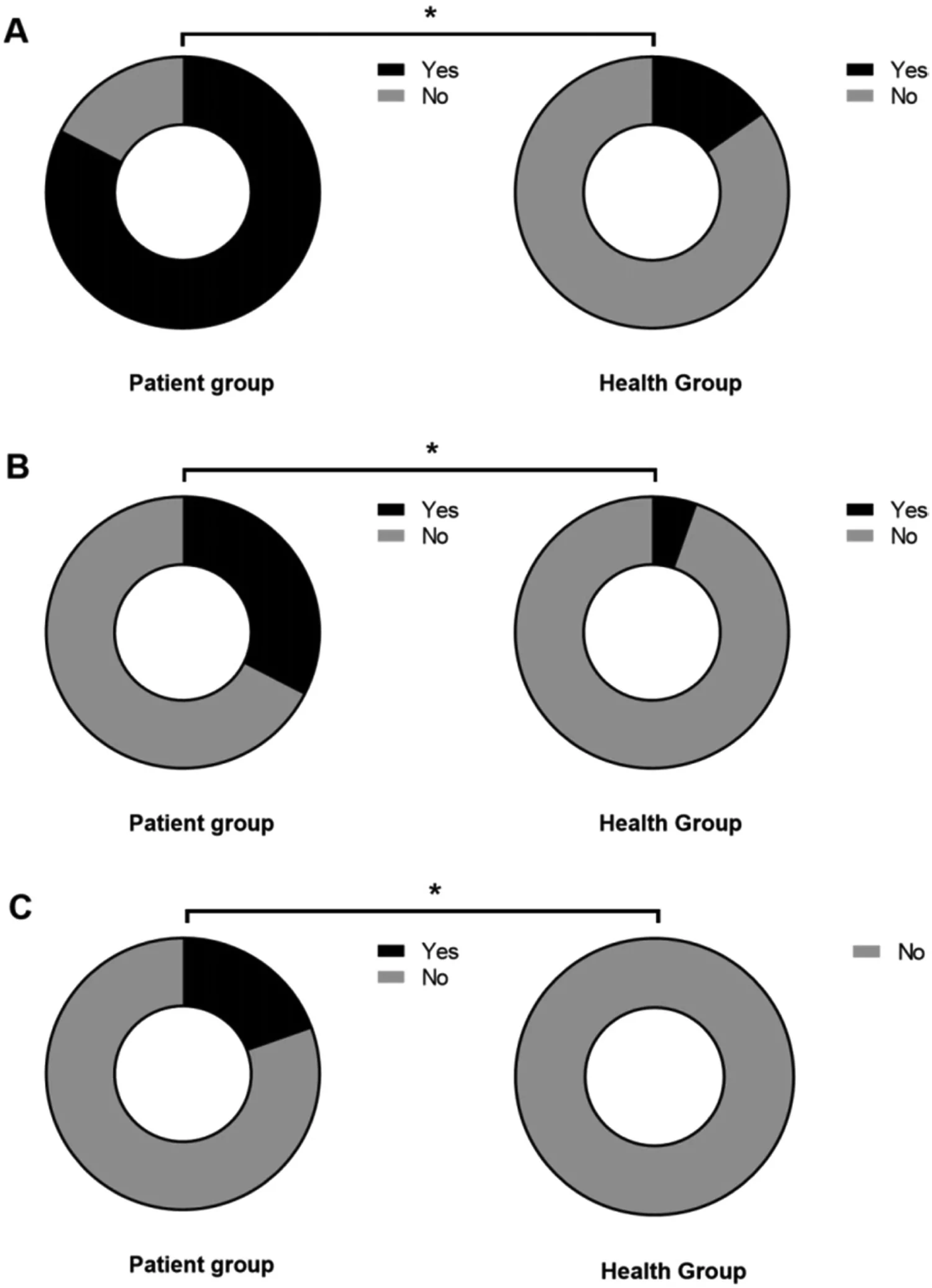
Differences in clinical manifestations between the two groups. **(A)** represents the comparison of the incidence rates of abdominal pain; **(B)** represents the comparison of the incidence rates of vomiting; **(C)** represents the comparison of the incidence rates of melena. * indicates that the difference in comparison is statistically significant, *P* < 0.05.

### 3.3 Laboratory indicators

The infection rate of Helicobacter pylori (63.0% vs. 8.7%) and the incidence rate of anemia (45.7% vs. 12.0%) in the child patient group were significantly higher than those in the healthy group. Gastroscopy showed that ulcers in the child patient group mostly occurred in the duodenal bulb (68.5%), followed by the gastric antrum (23.9%) and compound ulcers (7.6%). In Figure 3C, the category labeled “Other” refers to compound ulcers. See Figure 3.

**Figure 3:**
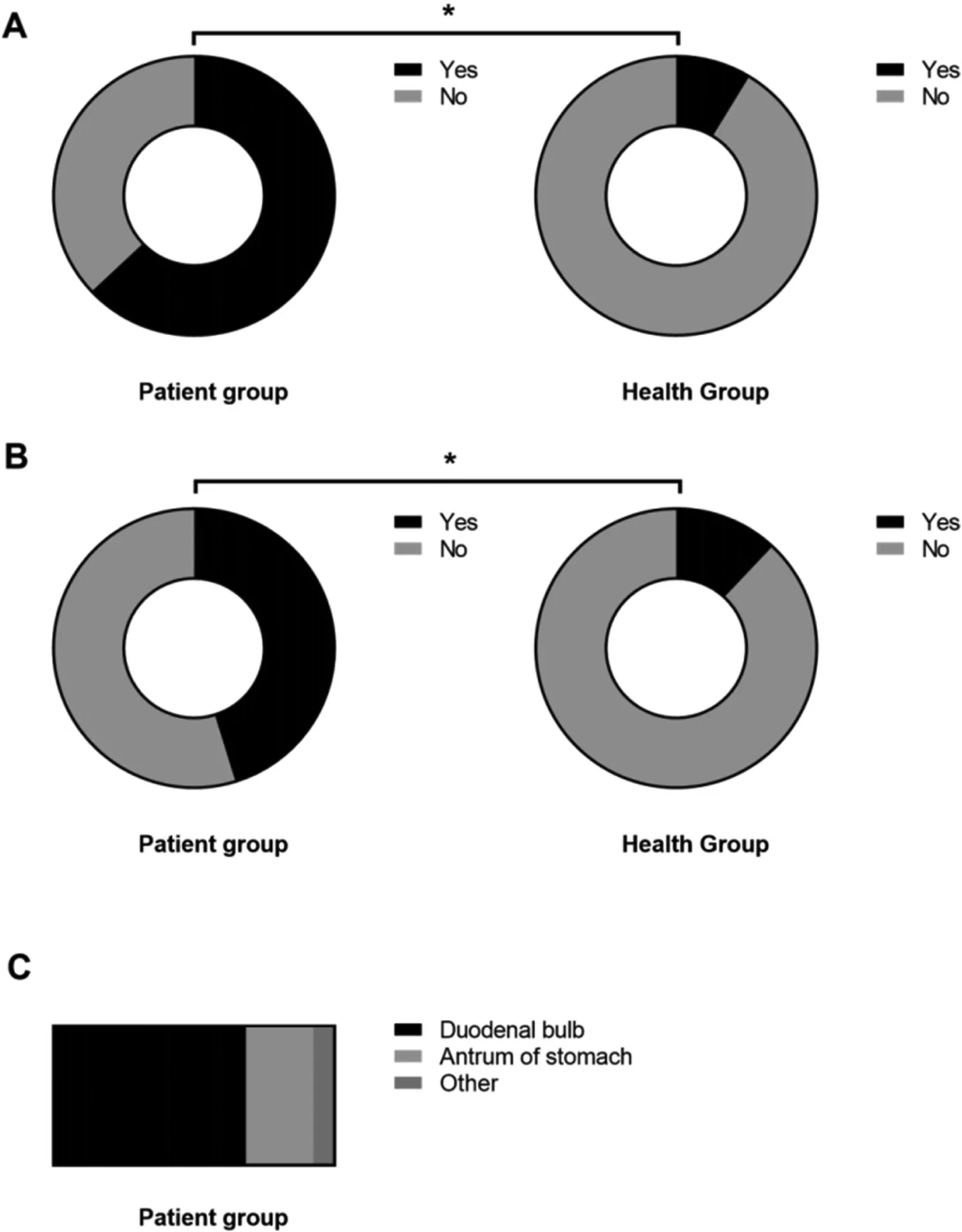
Comparison of laboratory indicators between the two groups and gastroscopic examination results in the child patient group. **(A)** represents the comparison of the infection rates of Helicobacter pylori; **(B)** represents the comparison of the incidence rates of anemia; **(C)** represents the distribution of ulcer sites in the gastroscopic examination results of the peptic ulcer group, including duodenal bulb ulcer, gastric antral ulcer, and compound ulcer. In panel **(C)**, “Other” refers to compound ulcer. * indicates that the difference in comparison is statistically significant, *P* < 0.05.

### 3.4 Lifestyle habits and family history

The proportion of children living at school (77.2% vs. 28.3%), the proportion of children with a high-sugar diet (56.5% vs. 28.3%), the proportion of children with a family history of digestive system diseases (30.4% vs. 8.7%), and the prevalence of psychological disorders (63.0% vs. 9.8%) in the child patient group were all higher than those in the control group (*P* < 0.05). In addition, the SCARED score was significantly higher in the peptic ulcer group than in the healthy control group (28.6 ± 8.4 vs. 14.2 ± 6.1, *P* < 0.05), indicating that anxiety-related emotional symptoms were more prominent in children with peptic ulcer. However, there were no significant differences in exercise habits and sleep duration between the two groups (*P* > 0.05). See Figures 4, 5.

**Figure 4:**
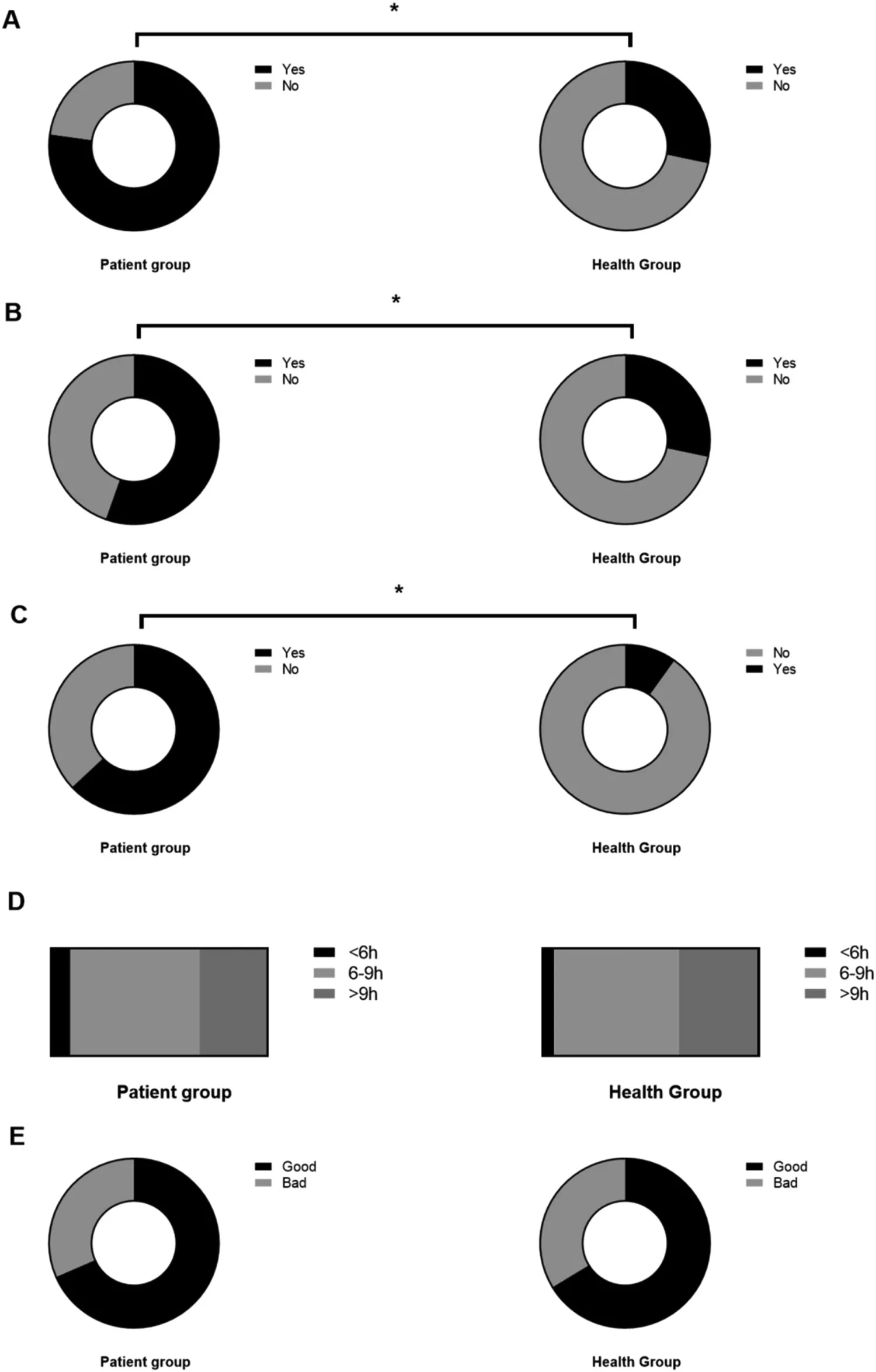
Differences in lifestyle habits between the two groups. **(A)** represents the comparison of the proportions of children living at school; **(B)** represents the comparison of the proportions of children with a high-sugar diet; **(C)** represents the comparison of the prevalence of psychological disorders; **(D)** represents the comparison of sleep duration; **(E)** represents the comparison of the rates of good exercise habits. *indicates that the difference in comparison is statistically significant, *P* < 0.05.

**Figure 5:**
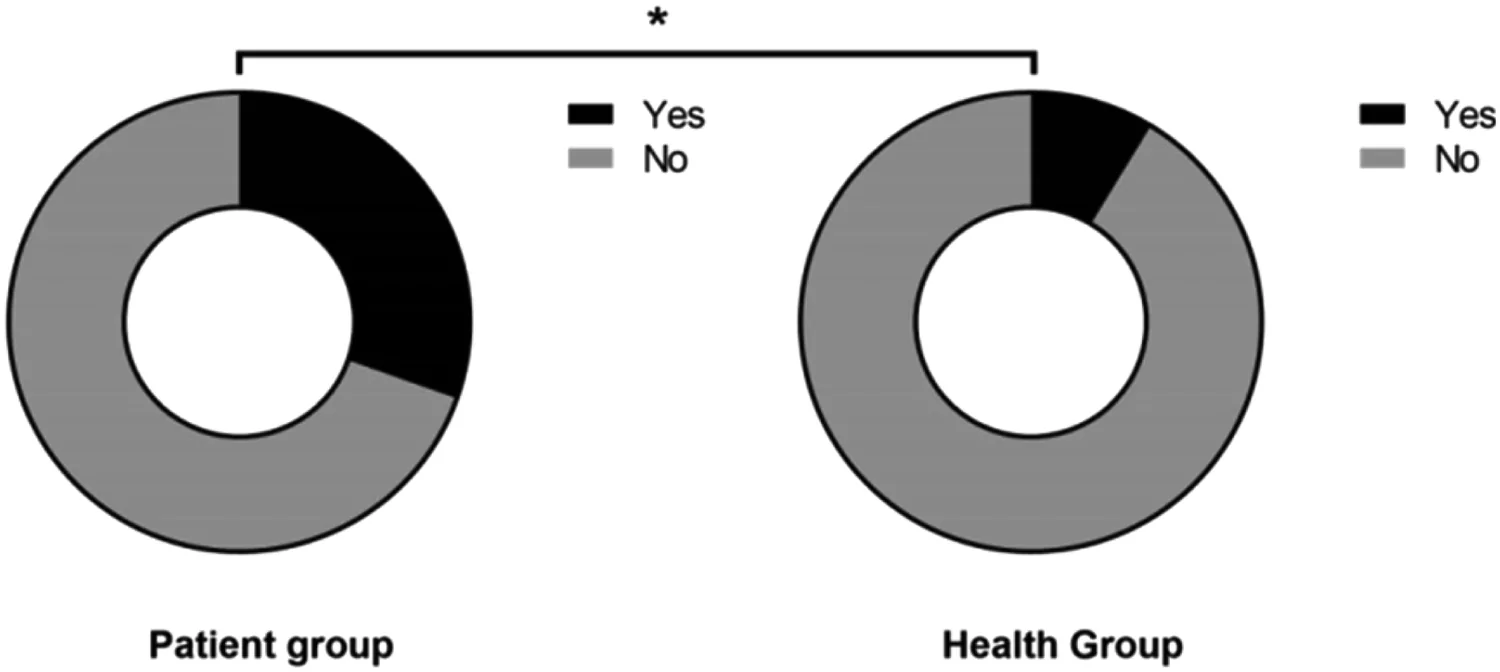
Proportions of family history of digestive system diseases between the two groups. *indicates that the difference in comparison is statistically significant, P < 0.05.

### 3.5 Multivariate analysis

Multivariate logistic regression analysis showed that Helicobacter pylori infection (OR = 4.23), high-sugar diet (OR = 2.89), family history (OR = 3. 17), living at school (OR = 2.41), and psychological disorders (OR = 6. 11) were independent risk factors for pediatric peptic ulcer (*P* < 0.05). See Table 2. After adjustment for multiple univariate comparisons using the Benjamini-Hochberg FDR method, the main between-group differences remained statistically significant. Candidate variables for the multivariate model were selected based on FDR-adjusted statistical significance, clinical relevance, and avoidance of variables that were likely to be consequences of peptic ulcer rather than pre-exposure risk factors. The Hosmer-Lemeshow goodness-of-fit test indicated acceptable model fit (*P* > 0.05). No obvious multicollinearity was observed among the independent variables, with all VIF values < 5 and tolerance values > 0.20.

**Table 2:** Logistic regression analysis of influencing factors in children with peptic ulcer.

Variables	*β*	SE	Wald *χ*^2^	*P*	OR	95% CI
Helicobacter pylori infection	2.07	0.78	7.43	<0.01	4.23	2.15–8.33
High-sugar diet	0.95	0.48	5.27	<0.05	2.89	1.42–5.89
Family history of digestive system diseases	1.28	0.55	6.21	<0.01	3.17	1.56–6.45
Boarding school attendance	0.91	0.51	5.88	<0.05	2.41	1.05–5.23
Psychological disorder	1.84	0.62	9.27	<0.05	6.11	2.61–10.94

OR, odds ratio; CI, confidence interval.

## 4 Discussion

This retrospective case-control study compared clinical manifestations, laboratory findings, lifestyle-related factors, family history, and psychological status between children with peptic ulcer and healthy controls. The principal findings were that Helicobacter pylori infection, a high-sugar diet, a family history of digestive system diseases, boarding school attendance, and psychological disorders were independently associated with pediatric peptic ulcer. These findings indicate that pediatric peptic ulcer may reflect a combination of infectious, lifestyle-related, familial, and psychosocial factors. Accordingly, the results support a multidimensional approach to clinical assessment; however, because of the retrospective case-control design, the observed associations should not be interpreted as causal. To place these findings in the context of previous pediatric evidence, we further compared our results regarding clinical manifestations, Helicobacter pylori infection, ulcer location, anemia, lifestyle-related factors, and psychological status with those reported in previous pediatric studies.

Firstly, the incidence rate of abdominal pain in the child patient group was significantly higher than that in the healthy group, a phenomenon closely related to the ulcer site and the release of inflammatory mediators. The duodenal bulb, as a high-incidence site for pediatric peptic ulcers, has a rich supply of nerve endings in its submucosa. After ulcer formation, it can directly stimulate pain-sensing nerve fibers, triggering upper abdominal or periumbilical pain. In addition, mediators such as prostaglandins and histamine released during the inflammatory response around the ulcer can enhance nerve sensitivity, leading to a decreased pain threshold (12–14). It is noteworthy that abdominal pain in the child patient group was mainly postprandial pain, which is associated with the peak period of gastric acid secretion and increased gastric antrum motility. After eating, gastric acid secretion increases, and the gastric acid that is not neutralized by food directly contacts the ulcer surface, exacerbating the pain. Moreover, increased gastric antrum motility may push gastric acid towards the duodenal bulb, aggravating local irritation. In contrast, the healthy group had a low incidence of abdominal pain, mostly functional abdominal pain, whose mechanism is more related to gastrointestinal motility abnormalities or visceral hypersensitivity rather than organic lesions.

The high incidence rates of vomiting and melena reflect the characteristics of complications of peptic ulcers. The significantly increased incidence of vomiting in the child patient group may be related to pyloric obstruction or delayed gastric emptying. When the ulcer is located near the pylorus, local edema or scar formation can lead to pyloric stenosis, causing mechanical obstruction, which is manifested by postprandial vomiting and vomitus containing bile. In addition, the excitation of the

vagus nerve caused by the ulcer can inhibit gastric antrum motility and delay gastric emptying, leading to symptoms such as nausea and vomiting (15, 16). Melena, as a typical manifestation of gastrointestinal bleeding, has a mechanism directly related to ulcer erosion of blood vessels (17, 18). Gastrointestinal bleeding in pediatric peptic ulcers often presents as occult blood loss or a small amount of hematemesis, which is related to the fact that children have thinner blood vessels and that bleeding is prone to self-coagulation. However, long-term chronic blood loss can lead to anemia, and the significantly higher incidence of anemia in the child patient group in this study is related to this mechanism. It is noteworthy that no melena occurred in the healthy group, which further confirms the clinical value of melena as a specific symptom of peptic ulcers. This finding is broadly consistent with previous clinical studies and reviews reporting that abdominal pain is the most common symptom of pediatric peptic ulcer, while vomiting and gastrointestinal bleeding-related manifestations are more frequently observed in children with more severe or complicated ulcer disease (5, 12, 17). However, because abdominal pain in children may also occur in functional gastrointestinal disorders, gastritis, or intestinal infection, clinical symptoms alone are insufficient for diagnosis, and further laboratory or endoscopic evaluation remains necessary when peptic ulcer is suspected. In a retrospective pediatric study by Ecevit et al., chronic abdominal pain was reported in 68% of children with peptic ulcer, gastrointestinal bleeding in 55%, vomiting in 39%, and Helicobacter pylori infection in 61% (19). The present study similarly identified abdominal pain as the most frequent clinical manifestation and found a comparable Helicobacter pylori infection rate, although vomiting was more frequent and melena was less frequent in our cohort. Differences in patient selection, disease severity, and symptom definitions may partly explain these variations.

Helicobacter pylori (Hp) infection, as a core risk factor for pediatric peptic ulcers, has had its pathogenic mechanism widely validated. In this study, the Hp infection rate in the child patient group was significantly higher than that in the healthy group, and multivariate regression analysis showed that Hp infection was an independent risk factor. Hp participates in ulcer formation through multiple pathways, including damaging the gastric mucosal barrier, promoting inflammatory responses, and affecting gastric acid secretion (20). Specifically, the urease produced by Hp can decompose urea to produce ammonia, directly damaging epithelial cells; the cytotoxin-associated gene protein it releases can induce epithelial cell apoptosis; and the cytokines released during the inflammatory response can activate immune cells, creating a chronic inflammatory environment (21, 22). In addition, Hp infection can also weaken the mucosal defense ability by affecting gastric mucosal blood flow and reducing mucus secretion. It is noteworthy that Hp infection is closely related to the recurrence of pediatric peptic ulcers. The high Hp infection rate in the child patient group in this study suggests the need to strengthen eradication therapy and follow-up management. Our result is consistent with previous studies showing that Helicobacter pylori infection is closely associated with gastric and duodenal ulcer formation through mucosal barrier injury, local inflammatory activation, and altered gastric acid secretion (20–22). Similar to previous findings, the present study further supports the importance of Helicobacter pylori detection in children with suspected peptic ulcer. Nevertheless, the association between Helicobacter pylori infection and pediatric peptic ulcer may vary according to region, age, and detection method, which should be considered in clinical interpretation. For example, Nguyen et al. (4) reported Helicobacter pylori infection in 93.8% of children with peptic ulcers, which was higher than the 63.0% observed in the present study. However, both studies showed a predominance of duodenal bulb ulcers, and the prevalence of anemia in their ulcer group was similar to that observed in our cohort. The difference in Helicobacter pylori positivity may be related to regional prevalence, referral patterns, and differences in diagnostic methods.

Gastroscopic examination results showed that ulcers mostly occurred in the duodenal bulb, which is related to the special anatomical and physiological characteristics of this site. The duodenal bulb mucosa lacks the protection of a mucus layer, and its

submucosa has a rich blood supply, making it vulnerable to gastric acid erosion. In addition, this site is in the peak area of gastric acid secretion, and gastric antrum motility can directly transport gastric acid to the duodenal bulb, creating a local high-acid environment. The gastric antrum, as the second most common site for ulcers, may have its ulcer formation related to Hp colonization, gastric antrum motility patterns, and relatively weak local mucosal defense ability (23–26). It is noteworthy that compound ulcers (affecting both the stomach and the duodenum simultaneously) accounted for a certain proportion in this study, suggesting that there may be a mechanism of multi-site involvement in ulcer formation, and comprehensive endoscopic examination should be paid attention to in clinical practice. The predominance of duodenal bulb ulcers in this study is comparable to previous reports indicating that duodenal ulcers are common in children with Helicobacter pylori-associated ulcer disease (23–25). This consistency suggests that the duodenal bulb may be a vulnerable site in pediatric peptic ulcer, possibly because of local acid exposure, mucosal susceptibility, and Helicobacter pylori-related inflammatory injury.

The roles of lifestyle habits and psychological factors in the occurrence of pediatric peptic ulcers cannot be ignored. The proportion of boarding children was significantly higher than that in the healthy group, which may be related to dietary hygiene, irregular daily routines, and psychological stress in the collective living environment. Boarding children often face issues such as a monotonous diet structure and irregular meal times, which may lead to disorders in the rhythm of gastric acid secretion. Moreover, differences in hygiene conditions in collective life may increase the risk of Hp infection. High-sugar diets participate in ulcer formation through pathways such as stimulating gastric acid secretion, affecting intestinal flora balance, and promoting inflammatory responses (8, 27, 28). In this study, the proportion of high-sugar diets was significantly higher than that in the healthy group, and multivariate regression analysis showed it to be an independent risk factor, suggesting that adjusting the diet structure may be an effective strategy for preventing pediatric peptic ulcers. The odds ratio (OR) of 3.17 for a family history of digestive system diseases, as a marker of genetic susceptibility, indicates that genetic factors play an important role in the occurrence of pediatric peptic ulcers. Existing studies have shown that certain gene polymorphisms (such as cytokine genes like IL- 1*β* and TNF-α) are associated with Hp susceptibility, mucosal repair ability, and the intensity of inflammatory responses, providing molecular biological evidence for the mechanism of action of genetic factors.

The mechanism of action of psychological disorders in pediatric peptic ulcers involves complex interactions of the “brain-gut axis”. In this study, the prevalence of psychological disorders in the child patient group was significantly higher than that in the healthy group, and multivariate regression analysis showed it to be the strongest risk factor. Psychological disorders (such as anxiety and depression) activate the hypothalamic-pituitary-adrenal axis and the autonomic nervous system, leading to increased vagal nerve excitability, increased gastric acid secretion, and abnormal gastrointestinal motility. In addition, psychological stress can also participate in ulcer formation through pathways such as affecting immune regulation, promoting inflammatory responses, and altering intestinal flora composition (29, 30). It is noteworthy that the action of the “brain-gut axis” is bidirectional—chronic pain and a decreased quality of life caused by peptic ulcers may further aggravate psychological disorders, forming a vicious cycle. Therefore, in clinical practice, it is necessary to pay attention to the psychological health assessment of children and, if necessary, combine psychological interventions to improve the prognosis. These findings are generally consistent with previous studies suggesting that dietary habits, irregular routines, psychological stress, and family susceptibility may influence gastrointestinal function through gastric acid secretion, mucosal defense, intestinal microbiota, inflammatory responses, and the brain-gut axis (8, 27–30). Compared with studies focusing mainly on Helicobacter pylori infection or endoscopic findings, the present study further highlights the potential role of lifestyle-related and psychosocial factors in pediatric peptic ulcer. This suggests that the prevention and management of pediatric peptic ulcer should not be limited to infection control, but should also include dietary guidance, lifestyle adjustment, family history assessment, and psychological support.

Although this study has revealed independent risk factors and differences in clinical characteristics of pediatric peptic ulcers, it still has certain limitations. Firstly, this study is a single-center retrospective study, which may be subject to selection bias and information bias. Although the healthy control children were selected from the same hospital and during the same period as the peptic ulcer group, they were recruited from children undergoing physical examination rather than from a community-based population; therefore, potential hospital population bias could not be completely excluded. Secondly, psychological assessment was conducted using scale scores rather than clinical diagnostic interviews, which may introduce measurement errors. In addition, this study did not include potential confounding variables such as environmental factors (e.g., air pollution, water quality), which may affect the comprehensiveness of the results. Future multi-center prospective studies should be conducted, incorporating a more comprehensive range of influencing factors and using more precise measurement tools to further validate the universality and reliability of the conclusions ofthis study.

## 5 Conclusion

In conclusion, the occurrence of pediatric peptic ulcers is the result of the combined effects of genetic, environmental, lifestyle, and psychological factors. In clinical

practice, a screening system for high-risk children should be established, with a focus on children with Hp infection, high-sugar diets, boarding status, and psychological disorders. Early interventions (such as Hp eradication therapy, diet structure adjustment, and psychological support) should be implemented to improve the prognosis. This study provides clinical evidence for the early identification and individualized management of pediatric peptic ulcer and has potential clinical application value.

## Data Availability

The original contributions presented in the study are included in the article/Supplementary Material, further inquiries can be directed to the corresponding author.
